# Changes in self-other boundaries modulate children’s body image attitudes

**DOI:** 10.3389/fnhum.2023.1181395

**Published:** 2023-05-03

**Authors:** Caryn Cook, Laura Crucianelli, Maria Laura Filippetti

**Affiliations:** ^1^Department of Psychology, Centre for Brain Science, University of Essex, Colchester, United Kingdom; ^2^Department of Neuroscience, Karolinska Institutet, Stockholm, Sweden

**Keywords:** enfacement illusion, self-face representation, multisensory integration (MSI), body image, self-other

## Abstract

One’s own face is a key distinctive feature of our physical appearance, yet multisensory visuo-tactile stimulation can alter self-other boundaries, eliciting changes in adult’s self-face representation and social cognition processes. This study tested whether changing self-face representation by altering self-other boundaries with the enfacement illusion modulates body image attitudes toward others in 6−11-year-old children (*N* = 51; 31 girls; predominantly White). Across all ages, congruent multisensory information led to stronger enfacement (η*^2^_*p*_* = 0.06). Participants who experienced a stronger enfacement illusion showed preference for larger body size, suggesting increased positive body size attitudes. This effect was stronger in 6–7-year-olds compared to 8–9-year-olds. Thus, blurring self-other boundaries successfully modulates self-face representation and body image attitudes toward others in children. Our results suggest that increased self-resemblance through self-other blurring resulting from the enfacement illusion may reduce social comparisons between self and other and result in positive body size attitudes.

## 1. Introduction

In his novel “One, No One and One Hundred Thousand” (Pirandello, 1990), the Italian writer Luigi Pirandello narrates the tragedy of Vitangelo Moscarda, a man that struggles to find his identity when he realizes the profound discrepancy that there exists between how he sees himself and how others see him. Moscarda’s identity crisis begins when his wife tells him that his nose tilts to the right, a facial feature that he had not noticed before. The sudden realization that other people “saw a Moscarda with a bent nose” becomes an identity dilemma, which leads to the recognition of a self that is fleeting (i.e., I am “no one”) and simultaneously dependent upon the perceptions of others (i.e., I am “one hundred thousand”).

Moscarda’s mirror experience in Pirandello’s novel highlights the intertwined and reciprocal link between social cognition and self-recognition. Across development, the ability to distinguish self from others plays a fundamental role in the emergence of self-awareness and allows infants to develop the skills necessary to engage in social interactions (Cooley, 1902; Mead, 1962; Rochat, 2009). Developmental research has demonstrated, for example, that the emergence of self-recognition in the mirror is associated with empathy (Bischof-Köhler, 2012) and pretend play (Lewis and Ramsay, 2004). Additionally, 16-month-old infants show motivation to align their own appearance to the appearance of others, suggesting that already at this young age, infants can engage in self-other comparison (Kampis et al., 2021). It has been suggested that, as self-recognition emerges, children gradually become not only aware of what they are, but also of the ways in which other people might perceive them (Cooley, 1902; Mead, 1962; Rochat, 2009). Given the high social valence of self-recognition, one might wonder how the boundaries between self and other are defined over time, thus allowing for a mental representation of the self-face to be acquired, maintained, and updated.

Research using on-line multisensory stimulation has demonstrated that it is possible to experimentally induce a partial overlap between one’s own representations of the self and another’s body. In the enfacement illusion (Tsakiris, 2008; Paladino et al., 2010; Sforza et al., 2010), it is possible to induce the experience of being in front of a mirror where, rather than seeing oneself, participants watch another person’s face. During the illusion, visual-tactile interpersonal multisensory stimulation (IMS) of the participant’s face that is synchronous and congruent with another person’s face leads to a change in self-identification, whereby the other person’s face is assimilated into one’s own representation through a process of self-other blurring (Tsakiris, 2008; Sforza et al., 2010; Tajadura-Jimenez et al., 2012a,b). While the enfacement illusion provides strong evidence of the malleability of the self-face representation, to our knowledge no studies have explored whether IMS in childhood produces a similar self-other blurring as the one evidenced by the adult literature. This is important because childhood is a developmental stage characterized by critical bodily, emotional, and cognitive changes that represent the scaffolding of a child’s sense of self and their social identity. Therefore, the first aim of this study was to examine whether IMS during the enfacement illusion alters self-identification in children (from 6 to 11 years old).

The experience of matching IMS provided by the enfacement illusion has been shown to affect different aspects of social processing. This is not very surprising, if one considers the role that faces play in interpersonal relations by allowing us to distinguish between self and other, but also between other and other (Zahavi and Roepstorff, 2011). For example, watching an avatar moving his head and touch his face synchronously with the participant leads to emotional contagion (Ma et al., 2016). Similarly, synchronous IMS increases facial mimicry (Minio-Paluello et al., 2020) and emotion recognition (Maister et al., 2013b). It has been proposed that increased self-resemblance induced by IMS is the mechanism through which social cognitive processes can be altered, suggesting that IMS-induced changes in self-representations may lead to a partial overlap between self and other, and thus change one’s perception of how physically similar others are in relation to oneself (Maister and Tsakiris, 2015).

Generalization of positive self-association induced by multisensory bodily illusions has been shown to extend beyond body-related aspects to the conceptual domain, whereby identifying with others affects attitudes toward other people (Maister et al., 2013a; Peck et al., 2013; Jeong et al., 2019). Relatedly, Salvato et al. (2020) showed that participants exposed to images of larger bodies are more likely to implicitly associate the concept of “self” with “thin,” suggesting that comparisons between self- and other-representations may modulate body image (Salvato et al., 2020). From a developmental perspective, this research is particularly relevant because examining the malleability of the self and the possibility of blurring the boundaries between self and other in development may shed light on the mechanisms underlying self- and social processing. In particular, the concept of body image as a mental picture of the appearance of a body (Cash, 2004) emerges during childhood, and it is strongly susceptible to social influences. Body image is a multifaceted construct that indicates thoughts, beliefs, behaviors, perceptions, and affective attitudes that individuals have toward their own body (Cash, 2004). Body image may also relate to attitudes individuals hold toward other people’s bodies (Paxton and Damiano, 2017). Research has demonstrated that body-related social comparisons are common among children and have been shown to trigger negative feelings and fears of deviating from a body norm (Neumark-Sztainer et al., 2006), and socially prescribed body ideals are already widespread and engrained well before the onset of puberty (for reviews, see Ricciardelli and McCabe, 2001; Smolak, 2004). In particular, research indicates that body image attitudes held in relation to the appearance of others start very early in life. For example, weight-bias against overweight children is already present in 3-year-old children (Spiel et al., 2012, 2016) and children between 6 and 11 years of age reported being the subject of weight-stigma in higher proportion if they were obese and overweight (compared to the normal-weight children) (Jendrzyca and Warschburger, 2016)—although this has been shown to be phenomena specific to WEIRD populations (Puhl and Heuer, 2009).

Overall, the abovementioned evidence suggests that body image attitudes toward other bodies require frequent comparisons between self- and other-representations. Thus, increasing self-other blurring, such as the one experienced as a result of the enfacement illusion, may have the power of altering body image attitudes. Supporting evidence comes from adult research showing that body image attitudes to the self can be changed via interpersonal multisensory stimulation (e.g., Preston and Ehrsson, 2014; Neyret et al., 2020). In addition, previous developmental research has shown that manipulating perceived body size leads to changes in subjective ownership, with illusory ownership over a larger, adult-sized rubber hand being successfully induced in children as young as 6 years of age (Filippetti and Crucianelli, 2019). With the present study, we examined—for the first time—the reverse effect, namely whether self-other blurring resulting from IMS can influence body image attitudes toward others in children aged 6−11 years.

Participants took part in an online experiment where we employed a modified version of the enfacement illusion to examine whether congruent vs. incongruent IMS between the self-face and a child-like avatar face would modulate body image attitudes toward the other, as measured using a revised version of the computer-based figure-choice scale (Evans et al., 2013; Mölbert et al., 2018). It has been suggested that the enfacement illusion relies on similar multisensory mechanisms as other bodily illusions, for example the rubber hand illusion (RHI, Botvinick and Cohen, 1998; Blanke, 2009; Tsakiris, 2010), whereby synchronous stroking between the participant’s own hidden hand and a visible rubber hand leads to a blurring between the two hands, with participants reporting the rubber hand being more similar to their own hand (Longo et al., 2009). Thus, in line with previous developmental findings in the context of the RHI (Cowie et al., 2013, 2016; Greenfield, 2015; Nava et al., 2017; Filippetti and Crucianelli, 2019; Gottwald et al., 2021), we hypothesized that congruent (vs. incongruent) IMS between the self and other face would elicit a change in the subjective experience of self-identification, with children across all ages reporting the experience of identifying oneself with the other face. Nonetheless, given that ours was the first study to examine changes in self-identification using the enfacement illusion, we deemed it important to explore the presence of developmental changes for at least two reasons. First, the face is arguably more distinctive of the self than the body (e.g., one’s own hand) and provides a number of cues allowing one to unambiguously distinguish the self from another person. Thus, it might be reasonable to expect from early to middle childhood differences in the ways in which children make use of such facial cues to discriminate self from other. Second, recent evidence suggests that social comparisons emerges around 5 years of age and increase across childhood (Wolf et al., 2021). It is possible to hypothesize that self-face representation would require a certain degree of plasticity to facilitate increasing comparisons between self and other. As such, one might expect to find a developmental progression throughout early and middle childhood. Considering the evidence from the adult literature suggesting that IMS can change social-related processes through a mechanism of increased self-resemblance (Maister and Tsakiris, 2015), we also hypothesized that congruent (vs. incongruent) IMS would influence children’s body image attitudes. Specifically, we expected to see that participants would display a preference for a larger body after being exposed to congruent IMS. We reasoned that, if the partial overlap between self- and other-representations experienced as a result of the enfacement illusion would increase likeness of the other, we should observe a reduction in social comparisons between self and other representations—and thus a decline in socially prescribed stereotypical body size attitudes. In other words, we expected that IMS between self and other would result in a reduction in weight bias (i.e., increased acceptance of larger body sizes). Given evidence that internalization of body image attitudes toward the self and toward others increase across childhood (Paxton and Damiano, 2017), we expected to see that positive body size attitudes due to IMS-induced changes would decrease from early to middle childhood.

Considering that the study took place during COVID-19, in secondary analyses, we sought to explore the mediating effects that physical distancing and social isolation due to the COVID-19 pandemic might have had on our effects of interest. We reasoned that this would be particularly important for the present study, given that emerging evidence suggests that the extended periods of “lockdown” during the pandemic, and the subsequent physical distancing and school closures, have had an impact on the mental and physical health of children and young people (Fegert et al., 2020; Wang et al., 2020), suggesting that this population is particularly vulnerable to social isolation. This is not surprising, as young people are driven to affiliate with their peer group and to be included with their peers (Gardner and Steinberg, 2005; Sebastian et al., 2010; Falk et al., 2014). Given the considerable physical growth and developmental changes that characterize childhood, the inability to engage in in-person social interactions with peers and the increased isolation during periods of lockdown could have affected how young people relate to others, as well as the self. Thus, it is plausible that any disruption to social relationships during this period can directly impact on the development of body and self-representations. Therefore, we asked our participants to also complete a short survey about their experience and feelings about being home from school (home-schooling) during the second UK national lockdown.

## 2. Materials and methods

### 2.1. Participants

Children were recruited through the Essex Babylab database, social media and word of mouth. Testing took place between January 2021 and April 2021, coinciding with the second UK national lockdown, during which UK children were being home-schooled. Before taking part in the study, the parent/legal guardian of each child had to complete an online form consenting for their child to participate. We tested fifty-one 6−11-year-olds (31 girls and 20 boys), mean age = 8.6 years, SD = 1.7 years. The sample was split into three balanced groups based on the children’s age (*N* = 17 6–7-year-olds, *N* = 17 8–9-year-olds, *N* = 17 10–11-year-olds). Using G*Power 3.1 (Faul et al., 2007), we calculated that in order to obtain a power greater than 0.80, with alpha set at 0.05 and *f* at 0.45, a total sample size of 42 participants was required (*N* = 14 for each age group condition). Our final sample was slightly larger than the recommended by *a priori* calculation because this is the first study investigating the enfacement effect in children. At the end of the experiment, participants were given the option to download a certificate of completion as a thank you for their participation in the study. Research was approved by the local research ethics subcommittee (ETH2021-0480).

### 2.2. Apparatus and stimuli

#### 2.2.1. Figure-choice task

To assess participants’ body image attitudes toward the other, we used a revised version of the computer-based figure-choice scale (Evans et al., 2013; Thaler et al., 2018) which utilized morphing images of age- and sex-matched avatar bodies. The avatar bodies were built using Make Human software. Both female and male avatar bodies maintained the default macros in terms of height, proportions and race (i.e., equal ratios of Black, Asian, and White features) and we chose the same clothes (“femalecausalesuit02”) for both sexes as this clothing was judged relatively unisex whilst also exposing the avatars’ limbs. Thinness and largeness of the bodies was selected by manipulating both the muscle and the weight features. Because the male bodies resulted in more built figures when increasing weight compared to the female bodies, we differentially manipulated the two sexes. For both female and male bodies, the thin avatar comprised of 39.90% muscle and 50% weight (which is the thin weight extreme set by the software for all avatars). With regard to the large bodies, the female avatar comprised of 20.90% muscle and 150% weight, whereas the male avatar comprised of 10% muscle and 150% weight (which is the large weight extreme set by the software for all avatars). This ensured that male bodies looked larger in terms of fat mass rather than simply more muscular. We created 12 different avatars, 6 (ages) × 2 (sex). All ages within each sex were manipulated in the same way. We implemented a computerized morphing procedure using Abrasoft Fantamorph 5 Professional to produce 10 body-morphing images, each with two different morphing directions, “thin to large” and “large to thin.” The figure-choice tasks (both in the pre-test and post-test) were run using Inquisit Web. Here, participants were instructed to choose the image that they liked the most by pressing a key. The same directions of morphing were used in the pre- and post-stimulation tests for each stroking condition, thus resulting in two trials for each figure-choice task. A figure-choice differentiation score was calculated by subtracting, for each participant, the mean pre-test from the mean post-test.

#### 2.2.2. Interpersonal multisensory stimulation (IMS)

**FIGURE 1:**
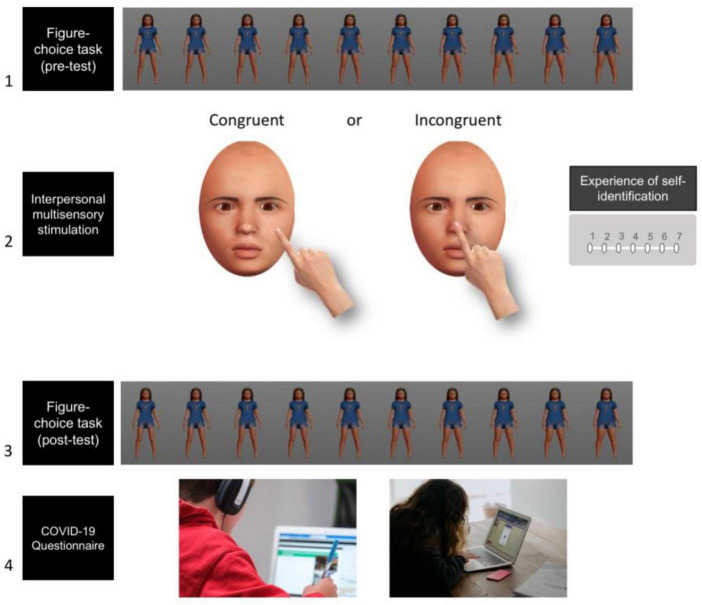
Illustrative example of the procedure of the study. The images in steps 1 to 3 display the avatar bodies and faces that the participants observed throughout the tasks. Participants underwent the pre-test figure-choice task **(1)** preceding the interpersonal multisensory stimulation (IMS) **(2)**, followed by the post-test figure-choice task **(3)**. The stroking mode (congruent vs. incongruent) during IMS was manipulated within-subjects, hence steps 1 to 3 were repeated twice. Finally, participants were asked to answer some questions about their home-schooling experience during the COVID-19 pandemic **(4)**. Photo on step 4 (left) by Compare Fibre on Unsplash. Photo on step 4 (right) by Annie Spratt on Unsplah.

To manipulate self-other boundaries and evoke a change in self-identification, we adapted IMS between one’s own and another person’s face during the classic enfacement illusion (e.g., Tsakiris, 2008) to ensure that children could perform the task online. During IMS, participants saw a gender-neutral avatar face (created using Make Human software—see Figure 1) being touched on the cheek every 6 s. The participant’s task was to touch either their own right cheek (congruent condition) or their own nose (incongruent condition) whenever they heard the sound “Touch.” IMS in each condition lasted 60 s. Given that we had little control over what the participants were doing during the tasks, we introduced a series of controls with the aim of reducing chances of inaccurate performance and/or misunderstanding of the task. First, given that experimenter couldn’t apply the tactile stimulation on the participants’ face herself, we asked participants to touch their own face every time they heard the sound “Touch.” This sound was always played 500 ms before the touch action seen on the screen and was introduced to reduce the likelihood of any time delay between the touch performed by the participants and the touch action displayed on the screen. This control over the timing of the touch is similar to the classic experimental protocol, whereby the experimenter’s tactile action is usually controlled by a metronome that ensures the touch is always applied at regular intervals.

Second, rather than manipulating visual-tactile synchrony as in the classic IMS paradigm used with adults (e.g., Paladino et al., 2010; Sforza et al., 2010; Tajadura-Jimenez et al., 2012a), we kept the timing of touch constant and instead manipulated the location of touch (i.e., congruency, in terms of which part of their face children were asked to touch, see Panagiotopoulou et al., 2017 for a similar approach). This was done to limit the possibility that participants could be misled by the touch seen on the screen and therefore touch their own face whenever they saw the action on the screen (i.e., even during the asynchronous condition). Therefore, in both conditions, participants saw a hand touching the avatar’s left cheek. The participant’s job was to either touch their own specular (right) cheek—in the congruent condition—or their own nose—in the incongruent condition.

The subjective experience of participants during each visual-tactile condition was assessed with 4 statements. Participants rated their agreement with each statement using a 7-point Likert scale (from “No, definitely not”; to “Yes, lots and lots”). The statements were based on previous adult studies that used the enfacement illusion and tapped into the experience of self-identification (Tajadura-Jimenez et al., 2012b). The statements were adapted as follow: (1) I felt like the other’s face was my face; (2) It seemed like the other’s face belonged to me; (3) It seemed like I was looking at myself in the mirror; (4) It seemed like I could move the other’s face.

#### 2.2.3. Questionnaires

Participants were asked to answer some questions (Qualtrics platform) in relation to their home-schooling experience during the national lockdown due to the COVID-19 pandemic. Specifically, the questions related to their feelings about (1) not being at school; (2) not being able to see their school friends; and (3) the idea of going back to school. Participants responded to these questions using a five-point Likert scale (from “Extremely sad” to “Not sad at all”). We also asked participants how often they used technology (FaceTime or Zoom, for example) to keep in touch with their friends during the lockdown. Participants responded to this question using a five-point Likert scale (from “Never” to “Almost all the time”). Finally, participants were presented with the Children’s Loneliness and Social Dissatisfaction (CLSD) Questionnaire (Asher et al., 1984). This scale comprises of 24 items, with 16 primary items designed to tap into children’s feelings of loneliness and social dissatisfaction in the school context. Participants responded to these questions using a five-point Likert scale ranging from 1 (not at all) to 5 (always).

### 2.3. Procedure

Participants underwent the pre-test figure-choice task preceding the IMS, followed by the post-test figure-choice task (see Figure 1). The stroking mode (congruent vs. incongruent) was manipulated within-subjects, their order counterbalanced across participants.

Before starting the experiment, participants were given a series of instructions about the ideal setup that would limit confounding factors affecting their performance. They were asked to sit comfortably and away from distractions, to ensure that the sound of their computer was on and to wear headphones to maximize attention throughout the experiment. After providing some demographics details (age and gender), they were shown a gender- and age-matched avatar and asked to select a name of their choice for the avatar.

Next, participants performed the pre-test figure-choice task. Participants were instructed to use the right and left arrow buttons displayed on the screen to move through the different avatar figures. Once they found the avatar that they liked the most, they had to click on the “Like” button on the screen. The task was repeated twice (one trial per morphing direction). Before the task, participants had the opportunity to practice one trial.

Next, participants underwent the IMS “induction movie” to elicit the enfacement illusion. It was explained to them that they would see a face of another child being touched by an index finger on the cheek and that sometimes they would also hear a voice saying “Touch.” Participants were then instructed to use their own index finger to touch their own right cheek (in the congruent condition) or their nose (in the incongruent condition) every time they heard the sound “Touch.” Before the task, participants had the opportunity to practice the task for 30 s. To avoid any carry-over effects, the practice trial consisted of visual-tactile asynchrony between the touch displayed on the screen and the touch applied by the participants. In other words, during the enfacement practice, the sound “Touch” was always played out of synchrony with the touch displayed on the screen. Therefore, as participants were asked to touch their own cheek whenever they heard “Touch,” during the practice trial we introduced a temporal asynchrony between the visual and tactile stimulation. This practice ensured that participants understood the instructions and were able to apply the touch on their face in response to the sound. After the IMS “induction movie,” participants were asked to rate their agreement and disagreement on 4 statements with regard to the illusion using a 7-point Likert scale (see Apparatus and Stimuli—Interpersonal Multisensory Stimulation).

Finally, participants performed again the figure-choice task as detailed above. Before repeating the tasks again (pre-test figure-choice task; enfacement illusion; post-test figure-choice task), participants were given the option of taking a short break. At the end of the second condition, participants were asked to answer some questions about being at home from school during the lockdown (see Apparatus and Stimuli—Questionnaires).

## 3. Results

### 3.1. Enfacement illusion

Questionnaire items were coded on a seven-point scale in response to questions about self-identification toward the avatar’s face. We applied Aligned Rank Transformation on the questionnaire data (Wobbrock et al., 2011). This procedure transforms non-parametric data (such as ordinal data from Likert scale from our questionnaire), into ranks (Conover and Iman, 1981). By undertaking an alignment step, the procedure then allows for statistically sound analysis of interaction effects, which would not have been possible with non-parametric tests see for similar statistical analyses (Cowie et al., 2018; Gottwald et al., 2021).

**FIGURE 2:**
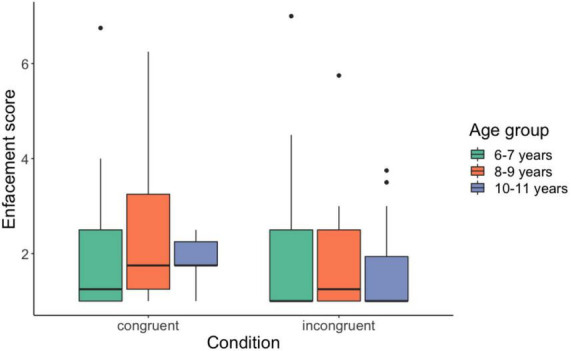
Mean enfacement scores for the three age groups. Means and standard errors across participants are shown.

We found significant main effects of Stroking Mode, *F*_(1,96)_ = 6.036, *p* = 0.015, η*^2^_*p*_* = 0.06, with higher values for congruency (Md = 1.75) than incongruency (Md = 1.25). No significant main effects of Age, *F*_(1,96)_ = 0.534, *p* = 0.588, η*^2^_*p*_* = 0.011, nor Age X Stroking Mode were found, *F*_(2,96)_ = 0.257, *p* = 0.773, η*^2^_*p*_* = 0.005 (see Figure 2). Further analyses at each enfacement statement separately, revealed that the difference between congruent and incongruent condition was driven by statement 3 “It seemed like I was looking at myself in the mirror,” *F*_(1,96)_ = 12.09, *p* < 0.001, η*^2^_*p*_* = 0.11. None of the other statements led to significant main effects [statement 1: *F*_(1,96)_ = 0.437, *p* = 0.510, η*^2^_*p*_* = 0.005; statement 2: *F*_(1,96)_ = 0.012, *p* = 0.915, η*^2^_*p*_* = 0.0001; statement 4: *F*_(1,96)_ = 0.505, *p* = 0.479, η*^2^_*p*_* = 0.005].

### 3.2. Figure-choice task

To analyze figure-choice data as a result of the enfacement illusion, we first ran a one-way ANOVA to examine whether pre-test scores at the figure-choice task differed among age groups. We did not find any difference in scores either in the first, *F*_(2,51)_ = 0.301, *p* = 0.741, or second condition, *F*_(2,51)_ = 0.365, *p* = 0.696. The lack of significant differences at baseline justifies the use of differentiation scores for the main analysis. Next, we performed a mixed 2 × 3 ANOVA, with Stroking mode (congruent vs. incongruent) as within-subject variable and Age (6−7 vs. 8−9 vs. 10−11) as between-subject variable. To account for multiple comparisons, we applied a Bonferroni’s correction to the reported *p*-values (i.e., *p*_corrected_ = *p*_uncorrected_/number of tests).

**FIGURE 3:**
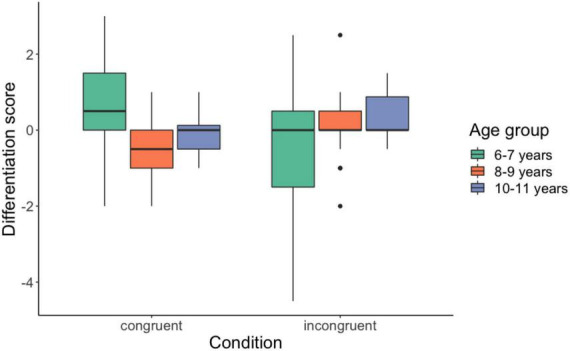
Mean differentiation scores for the figure-choice task for the three age groups. Means and standard errors across participants are shown.

We found no significant main effect of Stroking mode, *F*_(1,48)_ = 0.064, *p* = 0.801 and no significant effect of Age group, *F*_(1,48)_ = 1.944, *p* = 0.154. There was a significant Stroking mode X Age interaction, *F*_(2,48)_ = 4.156, *p* = 0.022. *Post-hoc* tests showed that, in the congruent condition, participants aged 6−7 years (*M* = 0.559, SD = 1.379) chose larger avatar figures compared to participants aged 8−9 years (*M* = −0.529, SD = 0.759), t(32) = 2.849, *p* = 0.008 (see Figure 3). There was no significant difference in differentiation scores between 6−7 vs. 8−9 age groups in the incongruent condition, t(32) = −0.880, *p* = 0.387. We did not find a significant difference in differentiation scores between 8−9 vs. 10−11 age groups in the congruent condition, t(32) = −2.356, *p* = 0.025 or in the incongruent condition, t(32) = −1.226, *p* = 0.229. Similarly, there was no significant difference in differentiation scores between 6−7 vs. 10−11 age groups in the congruent condition, t(32) 1.559, *p* = 0.129 or in the incongruent condition, t(32) = −1.768, *p* = 0.087. Finally, we did not find significant differences between congruent and incongruent conditions within the three age groups: 6−7 year olds: t(16) = 1.624, *p* = 0.124; 8−9 year olds: t(16) = −1.658; *p* = 0.117; 10−11 year olds: t(16) = −1.949, *p* = 0.069.

### 3.3. Correlation analyses

To test the hypothesis that increased self-blurring would be linked to a change in body image, we examined the association between figure-choice differentiation score and enfacement score across the three age groups. We found a significant correlation between figure-choice differentiation score and enfacement score in the congruent condition only. Specifically, participants who identified more with the avatar’s face during congruent IMS, chose a larger figure in the figure-choice task, *r* = 0.270, *p* = 0.028. The association between figure-choice differentiation score and enfacement score in the incongruent condition was not significant, *r* = 0.140, *p* = 0.163.

To explore the association between our dependent measures (figure-choice differentiation score and enfacement score) and the experience of the national lockdown due to COVID-19 on children’s social relationships, we conducted secondary correlation analyses. Responses on the primary items of the CLS were summed. Children’s scores on the CLS could range between 16 and 80, with higher scores reflecting higher degrees of loneliness. These analyses were conducted on a subset of participants that completed the COVID-19 survey (*N* = 43) and *p*-values were corrected for multiple comparisons using Bonferroni’s correction. We did not find any association between the CLS and our dependent measures. Similarly, we did not find any association between the questions pertaining to participants’ home-schooling experience and our dependent measures (see summary Table 1).

**TABLE 1:** Secondary correlation analyses examining the association between the study’s dependent measures (figure-choice differentiation score and enfacement score) and children’s home-schooling experience during the national lockdown due to COVID-19 pandemic.

	Differentiation scores congruent	Differentiation scores incongruent	Enfacement scores congruent	Enfacement scores incongruent
How do you feel about not being at school?	−0.037 (0.814)	0.262 (0.09)	−0.053 (0.736)	−0.062 (0.693)
How do you feel about not being able to see your school friends?	−0.123 (0.433)	0.228 (0.142)	0.01 (0.948)	0.1 (0.525)
How does the idea of going back to school make you feel?	0.05 (0.748)	−0.026 (0.87)	0.183 (0.24)	0.277 (0.073)
CLSD	0.145 (0.353)	−0.189 (0.225)	0.094 (0.55)	0.167 (0.285)

The table shows Pearson correlation and (*p*-values). CLSD, children’s loneliness and social dissatisfaction questionnaire.

## 4. Discussion

This study investigated whether congruent (vs. incongruent) IMS between the self and another person’s face modulates body image attitudes in 6−11-year-old children. In an online experiment, body image attitudes toward the other (according to body size) were assessed using a revised version of the figure-choice task (Evans et al., 2013; Thaler et al., 2018) before and after participants were exposed to congruent or incongruent IMS, aimed at eliciting the enfacement illusion (Porciello et al., 2018). As expected, we found that across all ages, the illusion was stronger during congruent than incongruent stroking. In addition, after congruent stroking 6−7-year-olds chose larger avatar figures compared to 8−9-year-olds, that is they showed higher preference for larger bodies. We also found that participants who identified more with the avatar’s face during congruent IMS, thereby exhibiting a stronger self-other blurring, showed a preference for a larger figure in the figure-choice task.

To our knowledge, this is the first study to directly investigate the possibility of manipulating self-other blurring in the context of self-face representation in children. We show that children aged 6 to 11 years of age reported a change in self-identification after congruent but not incongruent IMS, thus demonstrating that the enfacement illusion can be successfully induced in children as young as 6 years. These results show that the mental representation of what we look like is already flexible in childhood and can be updated as a result of bottom-up multisensory information. This is consistent with previous findings from research on body-parts other than face (e.g., hands), demonstrating that children use visual-tactile spatiotemporal information to establish and update own body representations (Cowie et al., 2013, 2016; Greenfield, 2015; Nava et al., 2017; Filippetti and Crucianelli, 2019; Gottwald et al., 2021). The current study adds to the existing developmental literature by showing that the same multisensory mechanisms that constrain body representations might be involved in updating the representation of one’s own face.

In our study, the effect of IMS was measured in terms of subjective change in self-identification. Specifically, we identified four statements from the adult literature (Tajadura-Jimenez et al., 2012b) that could provide a measure of change in self-other boundaries as a result of congruent and incongruent IMS. Our primary analysis demonstrated a significant main effect of congruent, over incongruent, IMS; however, we also show that the statement “It seemed like I was looking at myself in the mirror” was the key driver of the illusion. This result suggests that it was the illusory experience of looking at one’s own mirror reflection that the children in our study reported as being the strongest. Unlike the experience during bodily illusions that target the first-person embodied perspective, as in the RHI, the enfacement illusion requires the integration of first- (one’s multisensory experience) and third-person perspectives (the observed object in the mirror) (Tajadura-Jimenez et al., 2012b). During mirror encounters, the “I-subject” merges with the “me-object” recognized in the specular image, leading to the formation of a representation of one’s physical appearance that can be incorporated into a mental representation of identity. The results of our study demonstrate that, children identified with the face they see because the touch observed on the face was in the same location as the touch felt on themselves. In other words, by matching one’s sensory experience with the observed behavior, children in our sample identified with the avatar’s face displayed on the screen. Infant research has demonstrated that even 1-day-old infants are sensitive to multisensory visual-tactile stimulation between self and other face (Filippetti et al., 2013). We speculate that this early ability to detect contingencies between multisensory information by matching felt and observed sensory signals provides the scaffolding for the development of self-identification in childhood and may facilitate the formation of self-recognition abilities from early on.

The data reported here show that there were no age differences in IMS-induced changes in self-identification, thus suggesting that the strength of the illusion seems to remain stable across childhood. This finding is in line with RHI studies in children, which have demonstrated that the ability to identify a rubber hand as one’s own is relatively fixed between 6 and 10 years of age (Cowie et al., 2016; Nava et al., 2017). The finding that already from 6 years of age, the children in our study showed significant susceptibility to IMS-induced changes in self-identification may be surprising if we consider that changes in facial appearance (which may require frequent self-updating) seem to occur more significantly from adolescence (Bishara, 2000). However, the increasing availability of social media and technologies from early childhood means that children from a younger age are now able to represent and share the image of how they see themselves, or wish to be seen (Eichhorn, 2019). Thus, it is plausible that the greater active role that younger children nowadays play in representing themselves from their own perspective through the use of technologies requires a higher degree of self-updating to incorporate the multitude of selves that may reach a potentially wide audience. Future research should incorporate measures that directly assess children’s use of technologies and social media to examine their potential role in modulating self-representations from early childhood.

One of the aims of the study was to examine whether IMS-induced changes in self-identification would modulate body image attitudes toward the other. Specifically, we hypothesized that congruent, compared to incongruent, IMS would alter body image preferences by increasing the overlap between self- and other-representations (Maister et al., 2015). The resulting heightened self-resemblance would reduce self-other comparisons that frequently occur when evaluating bodies. Thus, we predicted that reducing such comparisons would lead to a reduction in socially prescribed stereotypical body size attitudes, meaning that participants would show a preference for larger bodies. We found that our hypotheses were partially supported by the data. Specifically, the increase in positive body size attitudes was evidenced in the 6−7-year-old children, when compared to the 8−9-year-old ones. However, we also found that overall participants who experienced a stronger enfacement illusion chose a larger figure (i.e., showed a preference for larger bodies in the post-test figure-choice task). These results are intriguing because they suggest that self-other blurring may reduce social comparisons between self and other representations and enhance the likelihood of generalizing positive self-associations to others (Maister et al., 2015). This effect has been largely evidenced in the adult literature, for example by showing that self-other overlap resulting from multisensory bodily illusion leads participants to rate others as more similar to themselves (Paladino et al., 2010), more trustworthy (Tajadura-Jimenez et al., 2012a), and better liked (Maister et al., 2013a). In the present study, we demonstrated that the same mechanism of increased self-resemblance through which IMS can change social related processes in adults, is already in place in early childhood. While we found a positive association between the strength of the enfacement illusion and changes in body image attitudes across ages, it is important to note that the specific effect in the avatar choice was only present when contrasting difference scores between the 6−7-year-old children and the 8−9-year-old children. Based on the literature on body image development, we can speculate the presence of a developmental trajectory that may account for differences in susceptibility to IMS-induced changes in body image attitudes. While body image attitudes toward the self and toward others have been increasingly reported at younger ages, internalization of these attitudes increase across childhood (Paxton and Damiano, 2017). For example, body satisfaction declines during late childhood (Clark and Tiggemann, 2006) and the desire to possess a thinner body increases with age (Dohnt and Tiggemann, 2006). Specifically, Dohnt and Tiggemann (2006) found that 6 years might be a critical age for the emergence of body image concerns, with the majority of children at this age wishing to be thinner than their actual size compared to their younger peers. While evidence on the development of body image is still scarce, we can speculate that in early childhood—with the emergence of body image concerns–children may have a heightened propensity to attend to others’ bodies, and in particular specific idealized bodies. This hypothesis is also corroborated by recent findings suggesting that, the tendency to underestimate how positively one is evaluated by others emerges around 5 years of age and increases across childhood (Wolf et al., 2021). This heightened social concern with other people’s evaluations of the self may lead to more frequent body-related social comparisons between perceived and ideal body at this age. In turn, this may require a higher degree of plasticity in self-representations, which can explain why we found a specific influence of IMS-induced changes in body image attitudes with the younger children. It is also possible that the absence of differences between our youngest (6–7-year-olds) and oldest (10–11-year-olds) groups may be due to the emergence of another stage of frequent body-related social comparisons beginning around puberty. As adolescence represents a critical period of vulnerability for the development and maintenance of body image concerns (Neumark-Sztainer et al., 2006), we hypothesize that this developmental stage may also be particularly vulnerable to IMS-induced changes in body image attitudes. While the present study did not include adolescent participants, future research should extend the present work to older children. Considering that body-related social comparisons have been linked to disordered eating attitudes and low self-esteem (Holt and Ricciardelli, 2002; Volpe et al., 2016), evidence of the presence of windows of susceptibility to IMS-induced changes in body image attitudes will have far-reaching implications for the development of prevention policies.

In the present study, we also included secondary measures to control for the potential effect of physical distancing restrictions and social isolation amid the COVID-19 pandemic on the development of self-representations. Participants were asked to complete the Children’s Loneliness and Social Dissatisfaction Questionnaire (Asher et al., 1984) as well as questions about their experience and feelings about being at home from school. We did not find a significant association between these measures and our dependent variables, thus suggesting that physical distancing measures and social isolation during the pandemic did not impact on self-face representation and body image attitudes, as measured in our study. We think, however, that this null result should be interpreted with caution, given that there is emerging evidence indicating that COVID-19 has had a significant psychosocial impact on children and adolescents (Duan et al., 2020; Fegert et al., 2020; Wang et al., 2020). In addition, school closure due to the pandemic might have increased the risk of weight gain and malnutrition among children (Rundle et al., 2020), with potential long-term consequences for the development of self-representations and body image attitudes. Thus, while our results may point toward non-significant outcomes, future research should longitudinally track the possible enduring effects that COVID-19 restriction measures have had on children’s development of the self.

Our study presents some caveats that can inform future work examining the association between self-other blurring and body image attitudes across the lifespan. These caveats primarily concern the adaptation of the experimental paradigm to be achievable in the online environment (compared to face-to-face in-person testing). In particular, with regard to the enfacement illusion, we implemented three main changes to the standard experimental setup. First, in the classic enfacement paradigm (see Porciello et al., 2018 for a review), participants experience touch delivered by the experimenter, which is synchronous and specularly congruent to the touch they see on the other person’s face. Given the experimenter’s physical absence during our study, participants had to touch their own face instead. Arguably, this adaptation from externally generated to self-generated tactile stimulation is closer to the naturally occurring encounters that individuals have with one’s mirror reflections, as when people touch their face in the mirror, the touch they feel is usually produced by themselves. Indeed, it has been shown that self-generated tactile stimulation during the synchronous IMS evokes comparable changes in self-face recognition as externally generated touch in adults (Tajadura-Jiménez et al., 2013). Building on this evidence, we demonstrate that self-touch can successfully elicit the enfacement illusion in children. Nevertheless, future work needs to implement IMS also using the standard externally generated touch condition, to examine its effectiveness in inducing the enfacement illusion in children. Second, compared to the standard enfacement paradigm where the two experimental conditions involve synchronous vs. asynchronous IMS, in the present experiment IMS between the self and avatar’s face was always delivered synchronously, and instead we manipulated the location where touch was delivered by participants. Hence, our main experimental manipulations involved congruent vs. incongruent, rather than synchronous vs. asynchronous, IMS [see (Panagiotopoulou et al., 2017) for a similar approach in adults]. While this multisensory manipulation differs from the standard setup, recent evidence from research on body-parts other than face show that children are susceptible to congruent vs. incongruent visual-tactile information during the RHI (Gottwald et al., 2021). Our findings corroborate and extend this evidence by showing that congruent IMS can induce the enfacement illusion in children, however, future studies should also contrast synchronous vs. asynchronous IMS. Third, to ensure that participants were applying the touch at regular intervals and in synchrony with the touch observed on the screen, we included a sound 500 ms before the touch action displayed. It could be argued that this audio input introduced an additional sensory component in the IMS task, whereby participants were exposed to audio-visual-tactile congruent and incongruent stimulation. We don’t think this has affected our results though, given that (1) the IMS was applied identically during congruent and incongruent conditions and (2) we show a significant effect of stroking mode. In sum, despite the aforementioned caveats, our study shows that the enfacement illusion can be successfully implemented in children using an online environment.

In addition, the adaptations implemented to run the study in the online environment mean that we could not check participants’ accuracy in performing the task, thus raising the concern that the emergence of the illusion might have been impaired. To help with this issue, we could have connected the experiment via webcam. Previous adult studies have shown that being recorded or observed leads to changes in body awareness (Baltazar et al., 2014; Durlik et al., 2014). Therefore, including such control would have also added a significant confound to our experiment as any change in self-identification could have potentially been attributed to being watched. Nevertheless, future research should aim to replicate our results in a laboratory setting. This would be important to ensure that confounding factors and task difficulty can be minimized in a well-controlled experimental environment.

It could be argued that, as we did not measure participants’ perception of the enfaced avatar, we have no means of knowing the extent to which children in our sample liked the avatar. This may be relevant given that our hypothesis was that enfacing an unfamiliar other would induce children to generalize positive self-associations to others. Nevertheless, previous adult studies using multisensory illusion that did not include measures of “affect” (e.g., perceived likeness/similarity of the embodied other) have been successful in demonstrating the effect of blurring self-other boundaries on social processes (e.g., Maister et al., 2013b; Jeong et al., 2019; Zhang et al., 2021).

In the present study we specifically manipulated body image attitudes toward the other. It would of course be fascinating to study how manipulating self-other boundaries modulates body image attitudes to the self, however, given the online setup for the study, we were unable to reliably collect measures related to the actual body size of participants to contrast against perceived body size. Body image in children is hard to quantify (Paxton and Damiano, 2017) and experimentally controlled procedures involving in-person testing may be best-suited to explore this topic. Nevertheless, we acknowledge that our body image results cannot be generalized to the self, and that future studies needs to address the specific effects of IMS-induced changes in self-representations in modulating body image attitudes toward the self.

Finally, while we did not collect socio-demographic data from the participants, it is likely that our sample comprised of individuals from a predominantly WEIRD population (cf. Rad et al., 2018). We therefore have to be cautious with generalizing the results to children from different backgrounds.

To conclude, children between 6 and 11 years of age already show flexible updating of their own self-face representation, with congruent IMS being a key factor in determining the malleability of self-other boundaries. We also show that IMS-induced blurring of these boundaries has the power of altering body image attitudes toward the other through a mechanism of increased self-resemblance (Maister and Tsakiris, 2015) that reduces social comparisons between self and other and results in positive body size attitudes.

## Data availability statement

The raw data supporting the conclusions of this article will be made available by the authors, without undue reservation.

## Ethics statement

The studies involving human participants were reviewed and approved by the University of Essex Research Ethics Subcommittee. Written informed consent to participate in this study was provided by the participants’ legal guardian/next of kin.

## Author contributions

MF and LC contributed to conception and design of the study. MF and CC set up the study and contributed to data collection. MF performed the statistical analysis and wrote the first draft of the manuscript. All authors contributed to manuscript revision, read, and approved the submitted version.
